# Falcarinol Is a Potent Inducer of Heme Oxygenase-1 and Was More Effective than Sulforaphane in Attenuating Intestinal Inflammation at Diet-Achievable Doses

**DOI:** 10.1155/2018/3153527

**Published:** 2018-10-21

**Authors:** Amanda L. Stefanson, Marica Bakovic

**Affiliations:** Department of Human Health and Nutritional Sciences, 50 Stone Rd E, University of Guelph, Guelph, ON, Canada N1G 2W1

## Abstract

Nuclear factor- (erythroid-derived 2) like 2 (Nrf2) is a transcription factor that regulates the expression of a battery of antioxidant, anti-inflammatory, and cytoprotective enzymes including heme oxygenase-1 (Hmox1, Ho-1) and NADPH:quinone oxidoreductase-1 (Nqo1). The isothiocyanate sulforaphane (SF) is widely understood to be the most effective natural activator of the Nrf2 pathway. Falcarinol (FA) is a lesser studied natural compound abundant in medicinal plants as well as dietary plants from the *Apiaceae* family such as carrot. We evaluated the protective effects of FA and SF (5 mg/kg twice per day in CB57BL/6 mice) pretreatment for one week against acute intestinal and systemic inflammation. The phytochemical pretreatment effectively reduced the magnitude of intestinal proinflammatory gene expression (IL-6, Tnf*α*/Tnf*α*r, Inf*γ*, STAT3, and IL-10/IL-10r) with FA showing more potency than SF. FA was also more effective in upregulating Ho-1 at mRNA and protein levels in both the mouse liver and the intestine. FA but not SF attenuated plasma chemokine eotaxin and white blood cell growth factor GM-CSF, which are involved in the recruitment and stabilization of first-responder immune cells. Phytochemicals generally did not attenuate plasma proinflammatory cytokines. Plasma and intestinal lipid peroxidation was also not significantly changed 4 h after LPS injection; however, FA did reduce basal lipid peroxidation in the mesentery. Both phytochemical pretreatments protected against LPS-induced reduction in intestinal barrier integrity, but FA additionally reduced inflammatory cell infiltration even below negative control.

## 1. Introduction

The gastrointestinal (GI) tract is the largest interface between the body and the environment, followed by the lung and the integument, with ratios of an estimated surface area approximately 150 : 50 : 1. The small intestine is the majority component of the GI tract; its surface was composed of a single monolayer of intestinal epithelial cells which secrete a glycocalyx matrix and a layer of mucous. This delicate barrier performs the diametric roles of digestion and absorption of nutrients and protection against pathogenic microorganisms and innumerable xenobiotic compounds from the environment [1]. In addition, the small intestine is the organ of first pass detoxification [2] and provides the milieu for a large proportion of the immune system [1, 3]. Likely due to this challenging physiological role, small intestinal epithelial cells have the highest turnover rates and are replaced every 2–6 days [4]. It is recognized that chronic and degenerative disease is rooted in early deviations from normal homeostasis that underpin the development of a wide variety of disparate disease pathologies. For example, unresolved inflammation contributes to cardiovascular disease, type 2 diabetes, metabolic syndrome, and neurodegenerative disease to name only a few. Intestinal barrier integrity is a lesser appreciated early deviation from homeostasis that contributes to many intestinal diseases (IBD, IBS, and celiac disease to name a few [5–7]) but also many other widely divergent pathologies. Barrier integrity has been implicated in autoimmune diseases, food allergies, obesity, endotoxemia, and chronic inflammation [5, 8, 9]. In fact, intestinal barrier function is very sensitive to seemingly unrelated traumas such as burn injury [10–12], hemorrhagic shock [13, 14], and even intense exercise [15–19].

Intraperitoneal lipopolysaccharide (LPS) is absorbed in the tissues of the peritoneal space, making its way into systemic circulation, where it is rapidly cleared from the bloodstream (minutes to hours [20, 21]) and slowly (over days [22, 23]) excreted from the organism in bile through liver metabolism, in the urine through kidney filtration, but also through the shedding of epithelial cells at the villus tip in the small intestine. The liver clears two thirds of circulating LPS via sinusoidal endothelial cells and Kuppfer cells [21], which is then secreted into the intestine via the bile [24]; in the lumenal environment of the intestine, there is a high tolerance for LPS due to the constant interaction with gram-negative bacteria in the microbiome [25, 26] and it does not trigger inflammation [27, 28]. LPS is ultimately excreted in feces [29, 30]. Some LPS loses occur via urinary excretion [30]. But another route of excretion is via the small intestine, where LPS appears first in the crypts and then concentrates in the small intestinal epithelial cells of the villus tips [31, 32], which are ultimately shed contributing another pool of LPS to fecal excretion. Intraperitoneal LPS causes shedding of small intestinal epithelial cells in a Tnf receptor- (Tnfr-) dependent manner within 1.5 hours at doses as low as 0.125 mg/kg [27]. The rapid manifestation of epithelial shedding, preceded by the crypt appearance of LPS, suggests transmigration of intraperitoneal LPS across the visceral peritoneum and not only derived from circulation. The amelioration of splenic injury from the introduction of normal mesenteric lymph into LPS-treated mice indicates a role for mesenteric fluids in systemic inflammation [33]. Intestinal clearance of LPS causes intestinal permeability, oxidative stress, and intestinal mitochondrial damage and increases lipid peroxidation [34].

**Figure 1:**
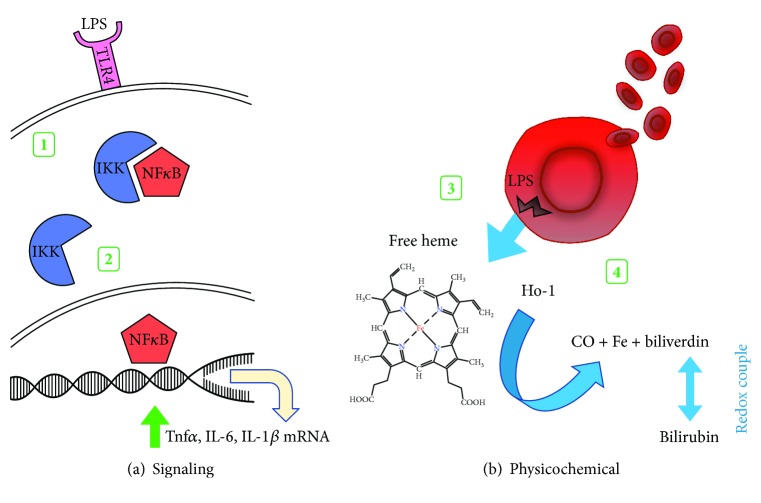
Effects of LPS. (1) LPS binding to the toll-like receptor-4 (TLR4) receptor initiates signaling to disrupt the inhibitor protein IKK association with proinflammation transcription factor NF*κ*B. (2) Free NF*κ*B translocates to the nucleus to increase the transcription of proinflammatory cytokines Tnf*α*, IL-6, IL-1*β*, etc. (3) LPS causes plasma membrane disruption in red blood cells releasing free heme. (4) Ho-1 breaks down free heme to equimolar amounts of CO, Fe, and biliverdin which is enzymatically converted to bilirubin, forming a redox couple.

**Figure 2:**
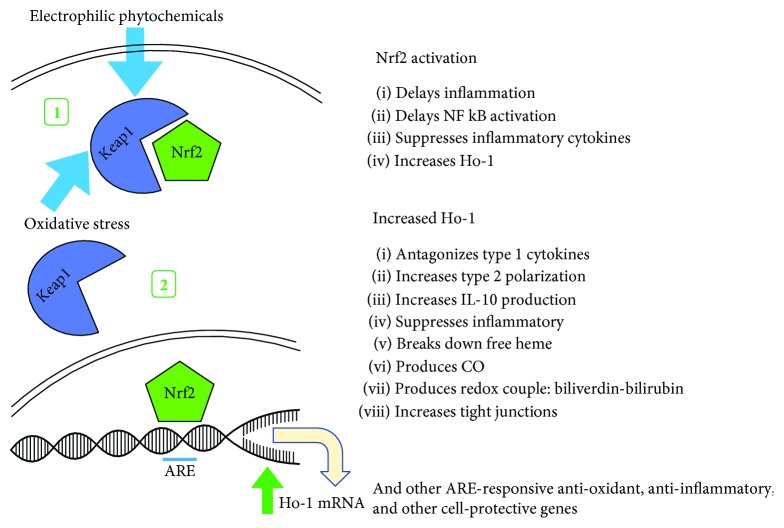
Effects of dietary electrophilic compounds on redox sensor Keap1. (1) Redox-sensing protein Keap1 is activated by intracellular oxidative stress or other electrophilic compounds, changing its conformation. (2) Keap1 releases transcription factor Nrf2 to translocate to the nucleus and upregulate the expression of a battery of antioxidant, anti-inflammatory, and cell protective genes including heme oxygenase-1 (Ho-1).

As shown in Figure 1, LPS initiates inflammation through toll-like receptor (TLR4) signaling that activates NF*κ*B-mediated cytokine production including Tnf*α*, IL-6 and IL-1*β* [35]. Keap1 is a redox-sensing cytosolic inhibitor protein for the transcription factor Nrf2 that upregulates the expression a battery of antioxidant, anti-inflammatory, and DNA repair genes including heme oxygenase-1 (Ho-1) [36, 37]. In response to increasing intracellular oxidation status or the binding of other electrophiles, the conformation of Keap1 is altered, releasing Nrf2 to translocate to the nucleus, binding the antioxidant response element (ARE) in the promoter regions of target genes (Figure 2) [38]. Priming the Keap1-Nrf2-ARE pathway with dietary electrophilic phytochemicals increases the threshold to the initiation of inflammation and delays the activation of proinflammatory transcription factor NF*κ*B [39–41]. The inhibitory role of Nrf2 has also been demonstrated in macrophages where it can bind ARE-independent DNA sequences in the promoter region of *IL-6* and *IL-1β*, suppressing their transcription [42]. Additionally, LPS can physically disrupt red blood cell membranes releasing free heme with prooxidant potential [43]. In its enzyme role, inducible heme oxygenase-1 (Ho-1) degrades free heme to equimolar amounts of carbon monoxide (CO), free iron, and biliverdin. Biliverdin is enzymatically converted to bilirubin which forms an antioxidant redox couple, while CO is independently anti-inflammatory [44]. Upregulating Ho-1 is protective against intestinal inflammation and loss of barrier integrity [45–47] and maintains alternatively activated/M2 macrophage polarization [48–50], shifting the polarization of intestinal T cells towards a regulatory phenotype [51–53].

Polyacetylenes are bioactive bisacetylenic phytooxylipins abundant not only in medicinal plants such as *Notopterygium incisum* (Qiang Huo) [54], *Angelica sinensis* (Dong Quai) [55, 56], and ginseng [57] but also in agricultural crops from the *Apiaceae* family [58], the most widely consumed of which is carrot [59, 60]. Falcarinol (FA) and falcarindiol (FD) are the most abundant carrot-derived polyacetylenes and have a demonstrated anti-inflammatory effect [60–62], in part by the suppression of NF*κ*B [63]. FD has been shown to activate Nrf2 by S-alkylation of its inhibitor protein Keap1 [64]. FD pretreatment upregulated the antioxidant enzymes NADPH:quinone oxidoreductase (Nqo1) and glutathione-S-transferase (GST), protecting against a later oxidative challenge in both normal liver cells [65] and an in vivo mouse model examining the activity of these enzymes in the liver, small intestine, kidney, and lung, in part by reducing lipid peroxidation [66]. Ginseng-derived panaxynol, structurally identical to carrot-derived falcarinol, is an anti-inflammatory compound and potent activator of cardiac Nrf2 [57]. In humans, panaxynol reduces oxidative stress-induced plasma lipid peroxidation [67]. We set out to evaluate for the first time the protective effect of diet-achievable levels of FA against intestinal inflammation in comparison to sulforaphane (SF)—widely recognized as the most potent natural compound activator of the Nrf2/ARE pathway.

## 2. Methods

### 2.1. Animal Treatment

**Figure 3:**
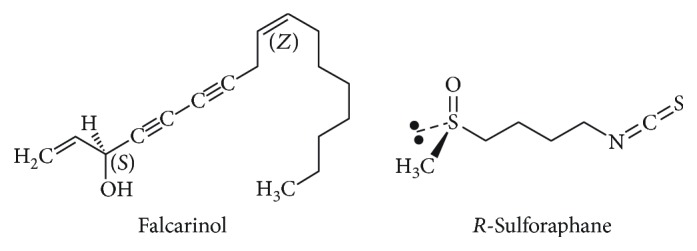
Structure of carrot-derived falcarinol and R-sulforaphane.

Three-month-old male CB57BL/6 mice (Charles River, St. Constant, QC, Canada) were individually housed in a temperature-controlled room on a reverse (12 : 12) light-dark cycle, fed a standard chow diet (Harlan Teklad, Mississauga, ON, Canada), with access to water *ad libitum*. Phytochemicals were prepared in 100% ethanol immediately before individual doses were prepared in peanut butter and allowed to evaporate overnight, refrigerated in a light-proof container. Twice per day for 7 days, 4 groups of mice received peanut butter (166 mg ± 0.01) with 5 mg/kg FA (CAS# 21852-80-2, Quality Phytochemicals LLC, East Brunswick, NJ, USA) (FA group), 5 mg/kg SF (CAS# 142825-10-3, Cayman Chemical, Ann Arbour, MI, USA) (SF group), or ethanol vehicle for the two control groups: a negative control (NC group) that was saline-treated and a positive control (PC group) that was lipopolysaccharide- (LPS-) treated. The chemical structures of FA and SF are shown in Figure 3. To elicit an immune response, the FA, SF, and PC groups of fasted animals (*n* = 3 per group) received an intraperitoneal injection of 5 mg/kg LPS on the eighth day and were sacrificed after 4 hours—a time point chosen for maximal intestinal inflammatory response [68, 69]. Plasma was collected by cardiac puncture, and tissues were removed and snap frozen in liquid nitrogen. All of the procedures conducted were approved by the University of Guelph Animal Care Committee and were in accordance with the guidelines of the Canadian Council on Animal Care.

### 2.2. Histological Analysis

Upper duodenal sections were flushed with saline and fixed in phosphate-buffered 10% formalin solution for 24 hours. Paraffin blocks were embedded, and 5 *μ*m sections in longitudinal orientation were slide-mounted, and haematoxylin and eosin (H&E) staining was performed by the Animal Health Laboratory at the University of Guelph. Histomorphological evaluation of H&E-stained slides was scored by a professional veterinary pathologist (Animal Health Laboratory-Kempville) in a blinded fashion using the methods outlined by Erben et al. [70]. Slides were evaluated for the inflammatory cell infiltrate score (as per Table 8, Erben et al.), and a number of mitotic cells were counted in 10 contiguous 400x fields [70].

### 2.3. Plasma Cytokines

Plasma cytokines were measured using a magnetic bead-based sandwich immunoassay according to the manufacturer's instructions (Bio-Plex Pro™ Mouse Cytokine 23-plex Assay, Bio-Rad Laboratories, Mississauga, Ontario). Antibody-coupled beads were incubated with plasma samples (1 : 3 dilution) in duplicate and incubated with biotinylated detection antibody to create a sandwich complex. Samples were subsequently incubated with streptavidin-phycoerythrin conjugate to serve as a fluorescent reporter. Beads were washed, and bound molecules were detected using a Bio-Plex 200 System (Bio-Rad Laboratories, Mississauga, Ontario).

### 2.4. PCR

mRNA was extracted from tissues using TRIzol according to the manufacturer's instructions (Thermo Fisher Scientific). mRNA concentration was evaluated by measuring absorbance using a Nanodrop spectrophotometer (Nanodrop 2000, Thermo Fisher Scientific). For each sample, 1 *μ*g of mRNA was incubated with DNase to remove genomic DNA and used for subsequent cDNA synthesis according to the manufacturer's instructions (iScript gDNA Clear cDNA synthesis kit, Bio-Rad Laboratories, Mississauga, Ontario). Resulting cDNA was amplified by real-time RT-PCR (CFX Connect, Bio-Rad Laboratories, Mississauga, Ontario) with select primers using PCR reagents according to the manufacturer's instructions (SsoAdvanced Universal SYBR Green Supermix, Bio-Rad Laboratories, Mississauga, Ontario). For each tissue, the geometric mean of 3 reference genes (*Rps29*, *18*s, and *Tbp*) was used to calculate the delta Ct for each gene of interest.

### 2.5. Immunoblotting

Tissue samples were homogenized in cell lysis buffer (liver) or RIPA buffer (intestine) and centrifuged at 15,000 *g* for 10 minutes at 4° C. Lysate supernatant was collected, and protein was quantified by bicinchoninic acid protein assay (Pierce Thermo Fisher Scientific) and measured with a plate reader (Molecular Devices, San Jose, USA). Protein concentration was standardized, and samples were separated in a 10% gel and transferred to PVDF membrane with a semidry electrophoretic transfer system (Bio-Rad Laboratories, Mississauga, Ontario). Membranes were incubated overnight with a 1 : 1000 dilution of primary antibody (Ho-1 and Nqo1, Abcam, Toronto, Canada), followed by a 1 : 3000 dilution of horseradish peroxidase-link secondary anti-mouse antibody (Cell Signaling Technology, Whitby, Canada). Blots were visualized with electrochemiluminescence reagent (Clarity Max, Bio-Rad Laboratories, Mississauga, Ontario), and images were captured with either FluorChem HD2 System (Cell Biosciences, San Jose, USA) or Gel Logic 6000 Pro (Carestream, Rochester, USA). Membranes were quantified using Image Studio™ Lite software (LI-COR Biosciences, Lincoln, USA) and normalized to either *β*-tubulin (liver) or total protein (intestine).

### 2.6. Lipid Peroxidation

Tissues were homogenized with 10 volumes of RIPA buffer containing protease and phosphatase inhibitors (Sigma-Aldrich P2714 and P5726, respectively) and centrifuged at 1600 *g* for 10 minutes at 4°C. The supernatant was used undiluted in the TBARS assay (Cayman Chemical, Ann Arbor, USA) according to the manufacturer's instructions. Briefly, the sample supernatant was combined with thiobarbituric acid assay reagents and boiled for 1 hour. Cooled sample preparations were loaded onto a 96-well plate and the absorbance read at 535 nm in a microplate reader (Molecular Devices, San Jose, USA), and lipid peroxides were interpolated from a malondialdehyde standard curve.

### 2.7. Statistical Analysis

Data were analyzed by one-way analysis of variance (ANOVA) followed by Tukey's posttest method to compare group means (“*P*” for ANOVA-derived *p* values and “*p*” for those derived from the posttest). All results with *α* < 0.05 were accepted as statistically significant; marginally significant results (*p* < 0.1; i.e., *α* < 0.10) are also mentioned. Qualitative scoring for intestinal inflammation was analyzed by Kruskal-Wallis one way analysis of variance, and Dunn's test was used to evaluate the pairwise mean rank difference. All data were analyzed with GraphPad Prism software (version 7).

## 3. Results

### 3.1. Falcarinol Was a Potent Reducer of Intestinal Inflammation

**Figure 4:**
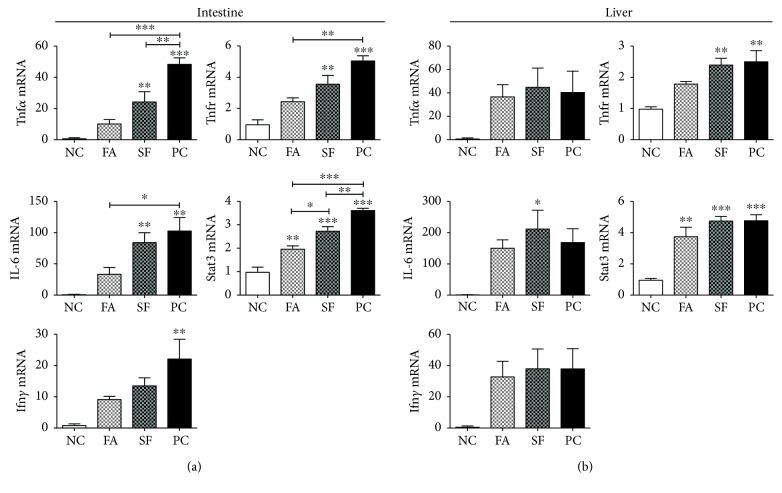
Intestinal and hepatic inflammatory gene expressions. Gene expression is expressed as mRNA fold change relative to negative control (NC). (a) Intestinal gene expression and (b) hepatic gene expression. Statistical significance is expressed as follows: ^∗^*p* < 0.05, ^∗∗^*p* < 0.01, ^∗∗∗^*p* < 0.001, and ^∗∗∗∗^*p* < 0.0001.

**Figure 5:**
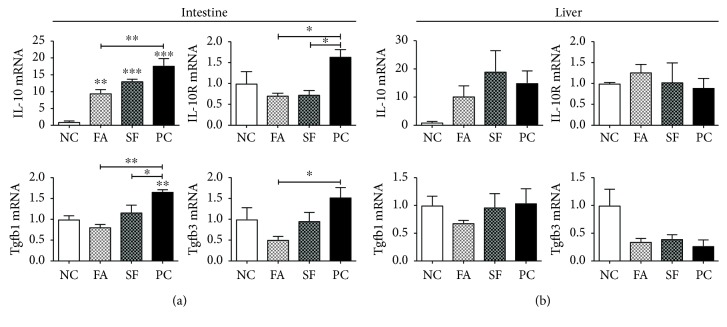
Intestinal and hepatic regulatory gene expressions. Gene expression is expressed as mRNA fold change relative to negative control (NC). (a) Intestinal gene expression and (b) hepatic gene expression. Statistical significance is expressed as follows: ^∗^*p* < 0.05, ^∗∗^*p* < 0.01, ^∗∗∗^*p* < 0.001, and ^∗∗∗∗^*p* < 0.0001.

Intestinal proinflammatory gene expression peaks between 4 and 6 hours after LPS injection [71], and maximal circulating proinflammatory cytokines Tnf*α*, IL-6, and IL-1 occur closer to 4 hours of postinjection [68, 72], so we chose the time point of 4 hours to best capture the acute intestinal and systemic inflammatory response. As shown in Figure 4(a), in the jejunum, both phytochemicals falcarinol (FA) and sulforaphane (SF) reduced the expression of LPS-induced proinflammatory genes, but FA was consistently more effective than SF. *IL-6* showed the greatest magnitude of change among the inflammatory genes, increasing 103.6-fold for LPS treatment alone (positive control (PC)) and 85.2-fold for SF (*p* < 0.01 for both), whereas for FA, the increase was prevented and the expression was not significantly different from the LPS-untreated negative control (NC). This pattern repeated for *Tnfα*, its receptor (*Tnfr*), and *Ifnγ*. *Tnfα* expression increased by 48.8-fold for PC (*p* < 0.0001) and 24.6-fold for SF (*p* < 0.01), and *Tnfr* was increased by 5.1-fold in PC (*p* < 0.001) and 2.6-fold in SF (*p* < 0.01), but for FA-treated mice, the increases were prevented and not significantly different from the negative control group. *Ifnγ* mRNA increased by 22.3-fold in PC (*p* < 0.001), but there was no significant increase for either phytochemical-treated groups. *Stat3* increased in all LPS-treated groups, increasing by 3.6-fold in PC, 2.8-fold in SF (both *p* < 0.0001), and 2-fold in FA (*p* = 0.0051). *Stat3* was significantly lower for FA than both PC and SF (*p* = 0.0002 and *p* = 0.0208, respectively). As shown in Figure 5, *IL-10* and its receptor (*IL-10R*) were also significantly increased by LPS treatment and the LPS response was reduced by both phytochemical treatments. *IL-10* expression was increased by 17.7-, 13.1-, and 9.5-fold in PC, SF, and FA, respectively (*P* < 0.0001), whereas its receptor increased only for PC (by 1.6-fold) and decreased with phytochemical treatment (both to 0.7-fold, *P* = 0.0176). Altogether, the phytochemical pretreatment effectively reduced the magnitude of intestinal proinflammatory gene expression with FA showing more potency than SF.

### 3.2. Phytochemicals Had a Minor Effect on Hepatic Inflammation

At 4 hours of post LPS injection, the effect on hepatic inflammatory gene expression was more subdued than in the intestine. As shown in Figure 4(b), the main inflammatory genes (*IL-6*, *Tnfα*, and *Ifnγ*) were all increased by LPS treatment and the phytochemical pretreatments showed no reductions in their expression. In fact, IL-6 had the greatest magnitude of increase for all LPS groups with the highest increase for SF (*p* = 0.0172). *Tnfr* mRNA was upregulated by 2.4-fold and 2.5-fold for SF and PC, respectively (both *p* < 0.01), that was presented in the FA group. *Stat3* was significantly upregulated in all LPS-treated groups (*P* = 0.0002). SF and PC both increased by 4.8-fold (both *p* = 0.0003). Differently from SF, FA caused the most conservative increase in *Stat3* (3.8-fold, *p* = 0.0022), showing some reducing effects.

### 3.3. Downregulated Expression of Intestinal Nrf2 Pathway Genes Was Not Rescued with Pretreatments

**Figure 6:**
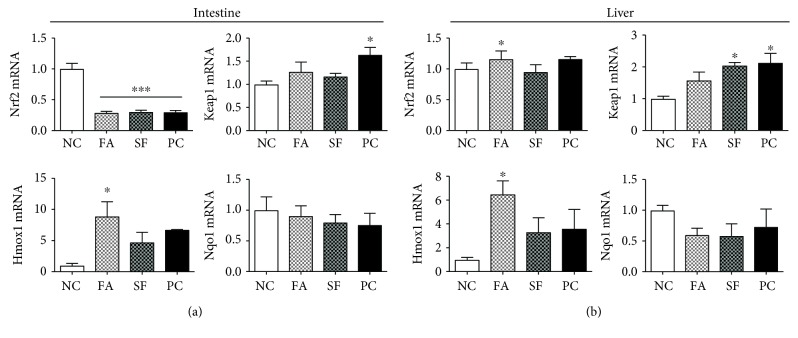
Intestinal and hepatic gene expressions of the Nrf2 pathway. Gene expression is expressed as mRNA fold change relative to negative control (NC). (a) Intestinal gene expression and (b) hepatic gene expression. Statistical significance is expressed as follows: ^∗^*p* < 0.05, ^∗∗^*p* < 0.01, ^∗∗∗^*p* < 0.001, and ^∗∗∗∗^*p* < 0.0001.

We also evaluated the effect of phytochemicals on the expression of Nrf2, Keap1, and their responsive genes *Hmox1* and *Nqo1* in both the intestine and the liver (Figure 6). In the intestine, the expression of *Nrf2* was 3-fold downregulated by LPS (*p* < 0.0001) and not rescued by phytochemical pretreatment at 4 hours of postinjection. *Keap1* was significantly upregulated only in PC by 1.6-fold (*p* = 0.045). We anticipated an increased expression of Nrf2 target genes by both phytochemical pretreatments but observed that only heme oxygenase-1 (*Hmox1*) was significantly increased only for FA by 8.9-fold above control (*p* = 0.0184). *Nqo1* (Figure 6) and *Muc-2* (not shown) gene expression also were not significantly changed by LPS with or without phytochemical pretreatments.

### 3.4. Falcarinol but Not Sulforaphane Stimulated Expression of Hepatic Nrf2 Pathway

In contrast, in the liver, LPS had no impact on the *Nrf2* pathway (Figure 6(b)). In fact, the FA pretreatment resulted in a significant increase of *Nrf2* mRNA (*p* < 0.05), and *Keap1* was significantly upregulated only in the SF and PC groups (2.0- and 2.1-fold, respectively; both *p* < 0.05). Similarly, *Hmox1* was significantly increased only in the FA-treated group (by 6.5-fold, *p* < 0.05), and there was no difference in expression between the SF and PC groups. *Nqo1* expression was not significantly affected by either LPS or phytochemicals in the liver.

### 3.5. Falcarinol but Not Sulforaphane Increased Intestinal and Liver Heme Oxygenase-1 Protein

**Figure 7:**
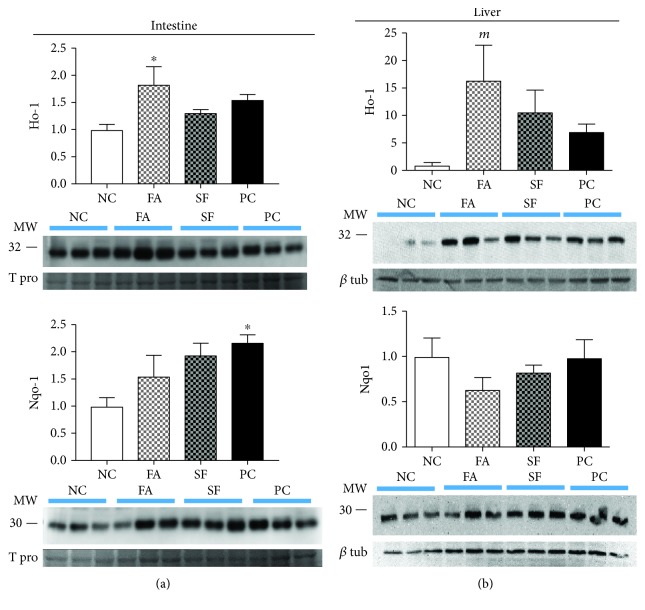
Intestinal and hepatic protein expressions of the Nrf2 pathway. Protein expression is expressed as fold change relative to negative control (NC). (a) Intestinal protein expression and (b) hepatic protein expression. Statistical significance is expressed as follows: ^∗^*p* < 0.05, ^∗∗^*p* < 0.01, ^∗∗∗^*p* < 0.001, and ^∗∗∗∗^*p* < 0.0001; marginally significant results (*^m^p* < 0.1) are also noted.

Interestingly, Ho-1 and Nqo1 proteins followed a similar expression pattern with respect to mRNA in both the intestine and the liver (Figure 7). The intestinal Ho-1 protein was significantly increased with FA (1.83-fold, *p* < 0.05), while there was no effect of SF or PC on Ho-1 protein. Similarly, the largest increase in hepatic Ho-1 protein was obtained only with FA (16.4-fold; *p* = 0.0806). On the other hand, the intestinal Nqo1 protein was increased with all treatments but was significant only in the LPS PC group (2.18-fold, *p* < 0.05). There was no effect of either phytochemical or LPS treatment on the liver Nqo1 protein.

### 3.6. Falcarinol Pretreatment Specifically Reduced Initial-Phase Plasma Cytokines

**Figure 8:**
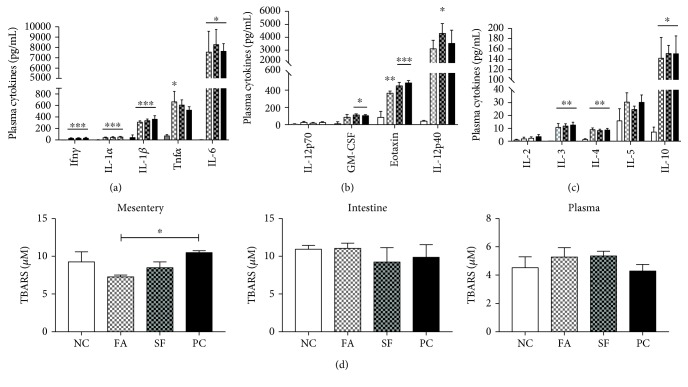
Plasma cytokines and lipid peroxidation. Circulating plasma cytokines 4 hours after LPS injection are expressed in pg/mL. (a) Classic inflammatory cytokines. (b) Other inflammatory cytokines. (c) Regulatory cytokines. (d) Lipid peroxidation measured by TBARS in the jejunal-associated mesentery, jejunum, and plasma 4 hours after LPS injection. Statistical significance is expressed as follows: ^∗^*p* < 0.05, ^∗∗^*p* < 0.01, ^∗∗∗^*p* < 0.001, and ^∗∗∗∗^*p* < 0.0001.

Eosinophils are the first immune cells to be recruited to the site of injury in response to locally produced eotaxin, and are followed by more numerous neutrophils and macrophages [73]. As shown in Figure 8, eotaxin increased 5.4-fold for LPS alone (*p* < 0.001, PC) and it was attenuated with FA, which only showed a 4.1-fold increase (*p* < 0.01), but not with SF (5.0-fold, *p* < 0.001) demonstrating that FA was able to show some protective effects in the initial phases of the LPS response. Granulocyte-macrophage colony-stimulating factor (GM-CSF) acts to recruit eosinophils and macrophages but is inhibitory to neutrophils [74]; GM-CSF was significantly increased only for SF and PC, by 5.6-fold and 6.7-fold, respectively (*p* < 0.05). IL-12p40 was significantly upregulated only for SF (by 90.2-fold, *p* < 0.05).

The plasma inflammatory cytokines (IL-1*α*, IL-1*β*, IL-6, and Ifn*γ*) were all significantly upregulated by LPS treatment, but there was no effect of phytochemical pretreatment on the magnitude of the response seen at the 4-hour time point. Tnf*α* was significantly increased only in the FA group (by 8.4-fold, *p* < 0.05). Other inflammatory factors IL-13, MIP-1*α*, and MIP-1*β* were significantly increased but without a protective effect of phytochemical treatment; similarly, the regulatory cytokines IL-3, IL-4, and IL-10 were all significantly upregulated by LPS with no effect of phytochemical pretreatment. All cytokines were increased in the plasma after LPS injection, with the exception of IL-9 which was not detectable in all samples. Changes were not significant for IL-2, IL-5, IL-17, KC, MCP-1, RANTES, or IL-12p70.

### 3.7. Falcarinol Specifically Reduced Lipid Peroxidation in the Mesentery

As shown in Figure 8, LPS had no significant effect on lipid peroxidation in the plasma, jejunum, or mesentery at 4 hours of postinjection; however, TBARS was significantly lower in the mesentery of the FA-treated mice (*p* < 0.05).

### 3.8. Falcarinol Completely Attenuated Inflammatory Cell Infiltration and Reduced Epithelial Turnover in the Intestine

**Figure 9:**
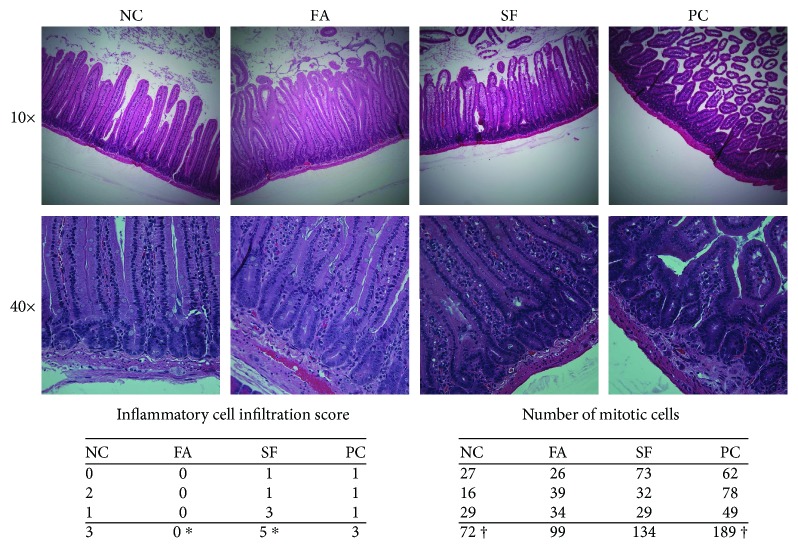
Histomorphological evaluation. Duodenal sections were slide-mounted and H&E stained. Histomorphological evaluation was performed blinded by a professional veterinary pathologist. Statistical significance is expressed as follows: ^∗^*p* < 0.05, ^∗∗^*p* < 0.01, ^∗∗∗^*p* < 0.001, and ^∗∗∗∗^*p* < 0.0001; marginally significant results are also noted (^†^*p* = 0.0522).

Qualitative scores for inflammatory cell infiltrate and epithelial damage were moderate 4 h after LPS treatment, ranging from 0 to 3 on a scale of 8. FA however completely attenuated LPS-induced inflammatory cell infiltration in the duodenum (Figure 9). Remarkably, despite LPS treatment, the score for FA was lower even than saline-treated NC. Both SF and PC scored similarly to NC; differences were only significant between FA and SF (mean ranks: 2.5 and 9.0, respectively, *p* < 0.05). The number of mitotic cells in the intestinal epithelium is a marker for the epithelial cell turnover rate [32, 75]. Results only approached significance between NC and PC (*p* = 0.0522). The mean numbers of mitotic cells counted in 10 contiguous 400x fields were 24, 33, 45, and 63 for NC, FA, SF, and PC, respectively (Figure 9). This study did not observe shedding directly, but histology revealed the architecture of PC duodenum to be so poor due to shortened villi (crypt : villus ratio is ~1 : 3 for PC as compared to 7–12 for the other groups) in which further morphological study was not possible. This effect was not seen in the LPS-treated groups that received phytochemical pretreatment.

## 4. Discussion

The anticancer effects of FA are its best characterized bioactive property [76–83]. FA also has positive metabolic effects. *In vitro*, FA improves insulin signaling in insulin-resistant porcine myotubes [84] and increases glucose uptake in normal porcine myotubes and adipocytes, as well as inhibiting adipocyte differentiation [85]. Interestingly, falcarindiol does not inhibit adipocyte differentiation but is a more potent PPAR*γ* agonist than FA which requires a higher dose to initiate an effect [54, 85]. FA stimulates normal intestinal cell growth at physiological doses, whereas carotenoids have no effect [86], and carrot juice has an anti-inflammatory effect in vitro intestinal cells [87]. FA also has anticomplement activity [88] and modulates GABA_A_ receptor activation [89].

In this study, we observed that the local effect of LPS on the intestine produced a greater response of inflammatory gene expression than in the liver, which would be expected to experience a lower dose of LPS derived from systemic circulation as opposed to directly from the intraperitoneal space. Additionally, the protective effect of the phytochemicals and falcarinol, in particular, was more pronounced in the intestine than in the liver. Intestinal cells would have been exposed to the full phytochemical dose over a short amount of time—a higher effective dose that would be available to cells relying on systemic circulation for phytochemical exposure such as the liver. The novel finding in this study is that falcarinol was more effective than sulforaphane in attenuating inflammatory gene expression in the intestine and to a lesser degree in the liver.

We also examined the effect of phytochemicals on Nrf2-activated targets, Ho-1 and Nqo1. Heme oxygenase-1 (Ho-1, *Hmox1*) has an emerging role in attenuating intestinal inflammation and protecting intestinal barrier integrity by upregulating the expression of tight junction proteins [47] and attenuating inflammation-induced intestinal permeability [46]. Prior Ho-1 upregulation protected intestinal barrier integrity by upregulating tight junction proteins, reducing apoptosis, activating Nrf2, and reducing NF*κ*B activation resulting from abdominal surgery in a rat model [90] and associated oxidative stress [91, 92]. FA, but not SF, significantly upregulated *Hmox1* in both the liver and intestine, whereas *Nqo1* expression was not affected by phytochemical. A unique characteristic of the *Nqo1* promoter is the number of ARE sequences. Rather than rendering *Nqo1* more Nrf2-sensitive due to the increased number of ARE contributing to its regulation, it appears that it is more likely that *Nqo1* requires more intense Nrf2 exposure to affect transcriptional activation than *Hmox1*. We demonstrated that *Hmox1* is more sensitive to Nrf2 activation than *Nqo1* in the liver and intestine which was also reflected in protein levels. Nqo1 protein levels were only significantly elevated in the intestine of PC, whereas in the liver, neither LPS nor phytochemical pretreatment showed an effect. Ho-1 protein levels were significantly increased from NC in both the liver and the intestine only in FA-treated mice, with no difference between the SF and the PC groups. Notably, this effect was only seen with FA-treated mice and not those treated with SF. This unique effect of FA is another novel finding in this study.

FA attenuated circulating eotaxin and GM-CSF (a proinflammatory inducer of M1 phenotype [93]) as compared to other LPS-treated groups, which could potentially translate to reduced immune cell recruitment, but cell trafficking was not evaluated in this study. Lipid peroxidation was not increased at 4 hours after LPS injection in any of the plasma, intestine, or mesentery; however, FA pretreatment reduced basal lipid peroxidation in the mesentery, which may be a contributing factor to the surprising reduction of inflammatory cell infiltration in FA duodena.

Glutathione-S-transferase is a phase II detoxification enzyme that conjugates electrophiles [94]. Sulforaphane is absorbed into intestinal cells as a glutathione conjugate [95], an interaction that is promoted by intracellular glutathione transferases [96] or a direct interaction with lumenal glutathione derived from the diet [97] or bile [98]. It is known that a portion of absorbed sulforaphane is secreted back into the intestine as a glutathione conjugate, reducing its bioavailability to 74% by some estimates [99]. The bioavailability of polyacetylenes has been demonstrated [100, 101], and they have been shown to bind human serum albumin for circulatory distribution [102], but we are not aware of any studies specific to their uptake mechanisms. Due to the electrophilicity of polyacetylenes; these mechanisms may be similar to SF, and possibly differential uptake efficiencies may contribute to the greater effectiveness of FA *in vivo.*

Normal epithelial cell loss from the villi tips is replaced by cells newly differentiated from crypt stem cells; a balance between cell loss and regeneration maintains intestinal barrier integrity. Accelerated mitosis in the epithelial layer is suggestive of shedding since there would be an increased need for regeneration to replace lost cells at the villus tip. LPS-treated groups had more mitotic cells than NC (1.38- and 1.88-fold more for FA and SF, respectively); PC had the most mitotic cells (2.63-fold more than NC) but did not reach significance (*p* = 0.0522), demonstrating the superior effect of FA over SF in protecting intestinal integrity. While LPS treatment did not substantively increase the qualitative score of inflammatory cell infiltration (mild to moderate infiltration in NC, SF, and PC), remarkably, FA did not show any infiltration despite LPS treatment (score = 0).

While it is possible that FA is a more potent activator of Nrf2 than sulforaphane, there may be other effects of FA that are responsible. Endocannabinoid signaling is involved in maintaining intestinal barrier integrity. Antagonism of cannabinoid type 1 receptor (CB_1_R) reduced intestinal inflammation and permeability in a diet-induced obesity model, attenuating metabolic endotoxemia and adipose inflammation and improving insulin resistance [103]. Pretreatment of the apical but not basolateral side of a Caco-2 cell monolayer prevented the cytokine-induced increase in intestinal permeability mediated by the antagonism of CB_1_R [104]. Dietary (apical side exposure) FA is likely protective of the intestinal epithelium since it is a covalent CB_1_R antagonist [105]; we are unaware of any studies directly evaluating sulforaphane for potential CB_1_R antagonism.

The current study evaluated the anti-inflammatory and antioxidant effects of isolated bioactive compounds available in the diet and their role in the prevention of inflammation (commonly understood to play an important role in the development of most chronic diseases) and more specifically in the context of intestinal inflammation and the maintenance of intestinal barrier integrity. Intestinal inflammation is particularly relevant since it provides the milieu for the polarization of naive T cells and other immune cells which have wider implications for the overall immune tone. The degradation of the intestinal barrier is gaining recognition as another early deviation from homeostasis contributing to the development of more serious and widely divergent diseases including some autoimmune conditions, food allergies, obesity, endotoxemia, chronic inflammation, and even intense exercise. Furthermore, our use of low/diet-achievable doses (5 mg/kg) as opposed to the commonly used default for studies of this type (100 mg/kg), which is a pharmaceutical or supplemental dose, make our findings all the more relevant since these effects are seen at dietary levels of exposure.

In conclusion, we have demonstrated the superior effectiveness of FA over SF at attenuating LPS-induced intestinal gene expression and to a lesser degree in the liver. FA was uniquely effective at upregulating Nrf2-target Ho-1 in both the intestine and the liver and attenuating some initial phase proinflammatory cytokines. FA also reduced inflammatory cell infiltration in the duodenum below even negative control and reduced basal mesenteric lipid peroxidation. These results suggest that the efficacy of FA may be fruitful to explore for prevention and treatment in inflammatory pathologies of the GI tract and in supporting the maintenance of intestinal barrier integrity due to the superiority of FA at upregulating Ho-1 to the anti-inflammatory and antioxidant effect demonstrated in the current study.

## Data Availability

The data used to support the findings of this study are available from the corresponding author upon request.
